# Advances in Label-Free Detections for Nanofluidic Analytical Devices

**DOI:** 10.3390/mi11100885

**Published:** 2020-09-23

**Authors:** Thu Hac Huong Le, Hisashi Shimizu, Kyojiro Morikawa

**Affiliations:** 1 Department of Applied Chemistry, Graduate School of Engineering, The University of Tokyo, 7-3-1 Hongo, Bunkyo, Tokyo 113-8656, Japan; 2 Collaborative Research Organization for Micro and Nano Multifunctional Devices (NMfD), The University of Tokyo, 7-3-1 Hongo, Bunkyo, Tokyo 113-8656, Japan

**Keywords:** nanofluidics, nanofluidic analytical device, label-free detection, microTAS, lab-on-a-chip

## Abstract

Nanofluidics, a discipline of science and engineering of fluids confined to structures at the 1–1000 nm scale, has experienced significant growth over the past decade. Nanofluidics have offered fascinating platforms for chemical and biological analyses by exploiting the unique characteristics of liquids and molecules confined in nanospaces; however, the difficulty to detect molecules in extremely small spaces hampers the practical applications of nanofluidic devices. Laser-induced fluorescence microscopy with single-molecule sensitivity has been so far a major detection method in nanofluidics, but issues arising from labeling and photobleaching limit its application. Recently, numerous label-free detection methods have been developed to identify and determine the number of molecules, as well as provide chemical, conformational, and kinetic information of molecules. This review focuses on label-free detection techniques designed for nanofluidics; these techniques are divided into two groups: optical and electrical/electrochemical detection methods. In this review, we discuss on the developed nanofluidic device architectures, elucidate the mechanisms by which the utilization of nanofluidics in manipulating molecules and controlling light–matter interactions enhances the capabilities of biological and chemical analyses, and highlight new research directions in the field of detections in nanofluidics.

## 1. Introduction

Over the past three decades, microfluidics and the integrated micro chemical systems on a chip have had a great impact on chemical analysis, synthesis, biosciences, and technologies [1,2,3]. Nanofluidics, the study of the behavior, manipulation, and control of fluids typically confined in structures of 1–1000 nm scale, currently attracts considerable attention for the development of new functionalities and applications. In nanofluidics, certain effects are expected to become significantly dominant due to the size reduction, such as laminar flow, diffusion, surface area to volume ratio, or surface tension. Nanofluidics is much more than the scaling down of microfluidics, because fluids on this size scale exhibit specific characteristics that are not observed on the microscale or in bulk. For example, water confined in nanospaces has unusual structural and dynamical properties such as higher viscosity, lower dielectric constant and refractive index, or higher proton mobility, compared to those in bulk [4]. Other confinement-induced effects are observed in terms of hydrodynamic flow, conductivity, and ionic transport. Furthermore, several ion transport phenomena that are absent or negligible in large microchannels become dominant in nanochannels, such as the localized enhancement of the electric field, the overlap of the electric double layer (EDL) that results in the ion selectivity [5].

Advances in nanofabrication and fluidic control techniques have stimulated the emergence of nanofluidic devices in bioanalyses. Various nanofluidic device configurations, including nanocavities, nanogaps, nanopores, nanopipettes, and nanoporous membranes, have been developed for bioanalysis purposes. For example, the physical confinement and denaturation of DNA molecules in nanofluidic channels has enabled the engineering of single DNA molecules [6]. Optical mapping of specific regions of interest was realized by exploiting the elongation of DNA molecules in nanofluidic channels [7,8,9]. Compared to conventional methods such as restriction mapping, optical mapping offers high-throughput analysis without enzymatic processing. The separation of DNA and large biomolecules using nanofluidics is also a topic of interest. A periodic structure of deep microfluidic and shallow nanofluidic channels was fabricated to create entropic traps for the size-dependent separation of DNA molecules [10], while an array of nanopillars was also used to separate long DNA molecules [11]. Utilizing the extremely large surface-to-volume ratio of the nanochannels, chromatographic separation was also implemented [12]. In addition, electrokinetic trapping at a micro/nanofluidic junction was conducted for the million-fold preconcentration of proteins and peptides [13]. Besides the novel functions originated from unique properties of fluids on nanoscale, confinement and localization of molecules in nanochannel also facilitate high-throughput single-molecule analyses as well as droplet and microwell techniques. For example, interactions and reactions of numerous biomolecules can be monitored without using confocal or total internal reflection microscopy [14].

The major obstacle that restricts the applications of nanofluidic devices in analyses is the difficulty in detection [15,16,17,18,19]. In general, the number of detected molecules in a nanofluidic channel is extremely limited. For example, when the concentration of an analyte is 1 μM, the number of analyte molecules in a 100 nm cube is 0.6 molecules. So far, the detection in nanofluidics mainly relies on the laser-induced fluorescence microscopy that offers single-molecule sensitivity. However, fluorescence involves a labeling process that causes various disadvantages including separation of unbound dyes, alteration of binding affinity or change in electrophoretic mobility, photobleaching or interference of fluorescence signals, and loss of quantitative response [20]. Moreover, the low labeling efficiency significantly impedes the detection of a single molecule or countable number of molecules in nanofluidic channels. Thus, there is an increasing demand for the development of label-free detections. Although the whole field of label-free detections is wide, beyond the scope of this review, state-of-the-art methods applicable or targeted to nanofluidics are summarized herein; these detection methods are mainly categorized into optical and electrical/electrochemical methods. The focus of this review is on the detection methods that not only enable in-situ and quantitative detection of molecules with high throughput, but also provide either chemical, conformational, or kinetic information of molecules. The nanofluidics-based manipulation of molecules and control of light–matter interactions to enhance the overall detection performance are particularly emphasized, and potential advances of label-free detections in nanofluidics toward biological and chemical analyses are discussed.

## 2. Optical Detection

In general, the detection of molecules in nanofluidic devices using conventional optical techniques is challenging, owning to their extremely short optical path lengths. For instance, the optical path length in a nanochannel is typically one millionth of that in a typical optical cell used in conventional absorbance measurements. One approach to detect the optical signals from a limited number of molecules in a nanofluidic channel is to realize background-free detection, in which no photon is detected by a photodetector or camera during the measurement of a blank sample. The background-free measurement is usually achieved by employing diffraction/scattering or differential interference contrast (DIC) techniques. Other major strategies involve the enhancement of light–matter interactions, which can be either temporal or spatial interactions, by using plasmonic and photonic structures.

### 2.1. Diffraction-Based Detection

Before the emergence of nanofluidics, many researchers have used nanochannel arrays as diffraction gratings. In particular, nanoimprinting lithography was widely conducted to develop polymer-based gratings, although most of the nanochannels in such gratings were not covered with top substrates. A nanofluidic grating, which is a sealed nanochannel array, was first reported in 2006 by Dumond et al. [21]. Later, Yasui et al. used the nanofluidic grating for determining the change in refractive index and for real-time monitoring of DNA amplification [22]. In their study, the diffraction efficiency for the diffracted light of the first order was monitored to detect the effective refractive index inside the nanochannels. Purr et al. prepared a device with asymmetric interdigitating arrangement of detection and reference nanochannels to directly calculate the refractive index [23]. This nanofluidic device could also be integrated into a smartphone-based biosensing system [24].

### 2.2. Scattering-Based Detection

Dark-field microscopy allows background-free sensitive detection of scattered photons from small particles [25]. Recently, interferometric detection of scattered light was reported and conducted for the single-molecule detection of non-labeled protein molecules [26]. This type of scattering-based label-free detection was also performed in nanochannels. For example, Mitra et al. used a heterodyne interferometer to detect viruses flowing through a nanochannel [27]. In addition, Faez et al. utilized a hollow-core optical fiber as a waveguide to introduce an incident beam, and photons scattered by metallic nanoparticles were detected in the dark field [28].

### 2.3. Plasmonic and Photonic Structure-Based Detection

Enhancement of light–matter interactions is a sophisticated technique to compensate for the reduced optical path lengths in nanofluidic devices. The spatial interaction between molecules and light, which apparently increases the optical path length, is improved in the total internal reflection or multiple reflection systems in wave-guided structures, cavities, photonic crystals, etc., while one can manipulate the temporal interactions of molecules and photons via plasmonic coupling in a plasmon resonant system. Plasmonics refers to a field in photonics involving the surface plasmon polaritons, which are the light-coupled coherent oscillations of free electrons at the interfaces of metals and dielectric materials. A plasmonic system can benefit from the inherent accumulation of optical field and energy, resulting in a significant enhancement of temporal interactions between light and molecules at a specific nanoscale space called the hot-spot [29,30]. The integration of plasmonic or photonic structures into a micro/nanofluidic channel to improve the detection performance has led to the emergence of optofluidics. The capacities of optofluidic devices have been further extended as combined with pretreatment processes such as chromatography and electrophoresis [31,32,33,34]. Optofluidic devices also enable novel optical functionalities such as optical forces to trap and manipulate particles or molecules [35,36].

Initially, plasmonic or photonic structures were typically embedded in microfluidic devices made of dimethylpolysiloxane (PDMS) and the microfluidic part was merely an additional accessory to introduce analyte molecules onto the sensing surfaces; this configuration was also referred to as “flow-over” apparatus. In such devices, the detection limits are often determined by the analyte (mass) transport limitations, rather than the detection capabilities of sensing structures. As a consequence, detection speed or the response time at low sample concentrations decreases significantly. Moreover, those “flow-over” devices suffer from the “dead sample volume” problem, because the detected volume, which is equivalent to the hot-spot volume, is much lower than the entire volume of microfluidic channel. Recently, the “flow-over” apparatus was replaced with a “flow-through” one by integrating plasmonic and photonic structures into physico-chemically well-controlled nanofluidic channels (i.e., optonanofluidic structure). In such systems, the nanofluidic channels represent the dielectric components of the photonic structures, thus maximizing the light–matter interactions while simultaneously leveraging the mass transport and overcoming the dead volume issue.

#### 2.3.1. Refractive Index Sensing

**Figure 1:**
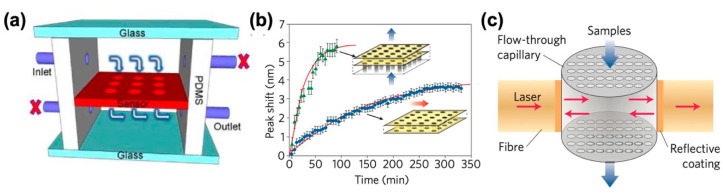
Nanofluidic devices using photonic structures for RI sensing: (**a**) nanohole array with “flow-through” scheme (adapted with permission from [37]. Copyright 2009 Optical Society of America), (**b**) the response time of sensors using plasmonic nanohole arrays with “flow-through” (green markers) and “flow-over” (blue markers) schemes showing a significant improvement in the response time of sensors in “flow-through” devices (adapted with permission from [38]. Copyright 2009 American Chemical Society), and (**c**) nanohole arrays integrated in a Fabry–Pérot (FP) cavity (adapted with permission from [42]. Copyright 2011 American Institute of Physics).

Refractive index (RI) sensors detect a subtle change in RI due to the homogeneous presence of analytes or their adsorption onto the sensing surface. Because the signal scales with the analyte bulk concentration or surface density rather than with the total number of molecules, RI sensing is less affected by the reduction of detection volumes in nanofluidic devices. Various nanofluidic device configurations using photonic structures, including nanohole-array-based photonic crystals (PhCs), plasmonic nanoholes as well as Fabry–Pérot (FP) cavities that exploit the simultaneous confinement of photon energy and molecules within a nanochannel, were found to be applicable for RI sensing and related practical applications. For example, Altug et al. employed a suspended membrane of PhC nanoholes (~10^2^ nm in diameter) integrated in a fluidic device for RI sensing purpose, as shown in Figure 1a [37]. The arrangement of inlets and outlets generates a convective flow through the PhC nanoholes, which directly introduces analytes into the active sensing sites of PhC to overcome the mass transport limitations. The same approach was demonstrated by employing plasmonic nanohole structures [38,39,40]. These configurations enable the rapid transport of target molecules to the hot-spots that significantly improves the analysis time, as shown in the sensor responses of the “flow-through” and “flow-over” schemes in Figure 1b. At least one order of magnitude improvement in mass transport and a detection limit of 10^−7^ RIU or pg mm^−2^ was confirmed. These devices were used in applications such as biorecognition binding and detection of a wide range of analytes—from protein molecules of several nanometers to viral particles of hundreds of nanometers. They are also promising for monitoring entire sample volume to enable “whole body” detection, which is particularly essential for single-cell analysis [41].

Another approach is utilizing the FP cavity to enhance the spatial light–matter interaction [42,43]. As illustrated in Figure 1c, a device consists of a micrometer-scale capillary with many built-in nanoholes (10^1^–10^2^ nm in diameter) placed between two reflectors, forming a FP cavity. The analytes binding to the internal surface of nanoholes are detected as a function of changes in the resonant wavelengths of the FP cavity. Note that the observed resonance cannot be attributed to the photonic bandgap effect resulting from the orderly arranged nanoholes but arises due to the intrinsic resonance of the cavity. The device exhibits a very high *Q*-factor and its “flow-through” configuration has contributed to the improvement of detection limit, compared to conventional FP cavity.

#### 2.3.2. Vibrational Spectroscopy

Vibrational spectroscopies, including IR absorption and Raman spectroscopies, are one of the most effective biochemical analysis methods, as they provide essential information on the chemical bonds and molecular structures in a label-free and non-invasive fashion. They are also useful in tracing subtle changes in the conformational structures of biomolecules in response to the surrounding environment or probing the kinetics of a chemical event. Raman and IR absorption spectroscopies are sensitive to different types of vibrational modes and provide complementary information about molecules. The former is sensitive to vibrations that alter polarizability, while the latter is sensitive to the ones that alter dipole moment. Even in a microfluidic device, the detection by Raman and IR absorption spectroscopies is challenging compared to UV/vis absorption or fluorescence emission spectroscopy, because Raman scattering and IR absorption are extremely weak processes. For example, only one in 10 million photons is Raman scattered [44]. Plasmon resonance on a thin film or nanoparticles and nanostructures of noble metals can significantly enhance the Raman signals (surface-enhanced Raman spectroscopy, SERS) of molecules by several orders of magnitude (10^10^ to 10^14^) through electromagnetic field enhancements [45]. Similar effects were also observed in IR absorption (surface-enhanced infrared absorption spectroscopy, SEIRA) [46,47]. These effects, however, are only observed when molecules are located in the vicinity of the hot-spots of the plasmon resonators; thus, positioning of target molecules exactly at the hot-spots is crucial to effectively utilize the plasmonic–molecular coupling. Despite recent efforts in this field, most of the studies focused on either engineering or tailoring the plasmonic structures, while molecules were randomly adsorbed or flowed over the sensing surfaces. In such cases, the small spatial overlapping of hot spots and molecules reduces sensitivity and reproducibility, thereby impeding the quantitative analysis.

##### Surface-Enhanced Raman Spectroscopy (SERS)

The efficiency of “flow-though” nanofluidic devices in improving the mass transport were confirmed for SERS. Oh et al. demonstrated a “flow-through SERS” device by using a suspended plasmonic nanohole array; they obtained a signal 50 times stronger compared to that achieved by using the “flow-over” device [48]. Similar “flow-through” configurations employing porous anodic aluminum oxide (AAO) [49] or opal photonic crystal capillaries decorated with metallic nanoparticles are also promising platforms for performing “flow-through SERS” [50].

**Figure 2:**
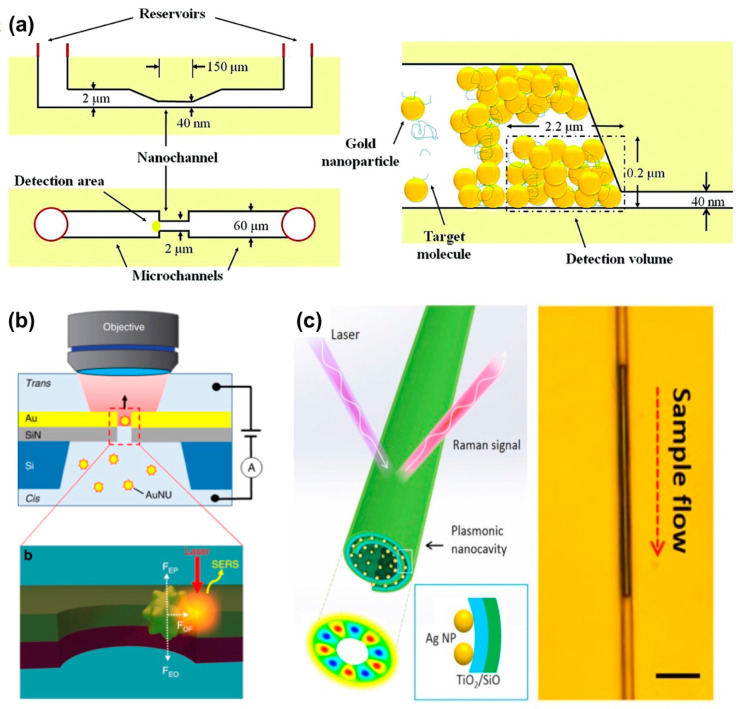
Nanofluidic devices for SERS: (**a**) device exploiting the localization of nanoparticles and preconcentration of target molecules at micro/nanochannel junction (adapted with permission from [51]. Copyright 2011 American Chemical Society), (**b**) electrokinetic capture of a single molecule at a nanopore to increase its residual time (adapted with permission from [55]. Copyright 2019 Springer Nature), and (**c**) self-rolled nanotube integrated with Ag NPs supporting the coupling of whispering-gallery resonances and surface plasmon generated on Ag NPs that improves the enhancement factor in SERS. The scale bar in the right image is 3 μm (adapted with permission from [56]. Copyright 2015 Springer Nature).

Another strategy to overcome the low sensitivity and low reproducibility of SERS is to employ a micro/nanochannel interface to exclusively preconcentrate the target molecules. Figure 2a shows a pinched and step microchannel–nanochannel junction that enables size-dependent trapping of gold colloid particles, creating gold nanoparticle clusters of high density. In a similar fashion, the capillary flow delivers the target molecules through the interstices among the clusters, increasing the concentration of the target molecule at the SERS hot-spots. This device configuration was implemented in various applications including diagnosis and understanding of Alzheimer’s disease by tracing the conformational transition of β-amyloid peptide [51,52]. In another attempt to decrease the “dead volume”, colloidal nanoparticles of ~100 nm in diameter were linearly arranged in the nanochannels by a self-assembly process. This configuration not only ensures that all analyte molecules flow through the hot spots of the nanoparticles but also enables a significant Raman enhancement by matching the polarization direction of the incident light to the alignment direction of particles [53].

In the “flow-through” SERS, molecules pass the detection point so rapidly that the collected SERS signals are not sufficient for single-molecule analysis. By employing a single nanopore [54] or nanoslit [55] that allows the electrokinetic capture (i.e., electroplasmonic trapping effect) to increase the residual time of target molecules within the hot-spots, as illustrated in Figure 2b, it is possible to trace the spectroscopic fingerprinting of a single DNA molecule. The device has also been applied in detection of all four DNA bases in a single DNA molecule, as well as in identification of single nucleobases in a single oligonucleotide. The above-mentioned results indicate the capability of nanofluidics in manipulating single molecules, as well as the light–matter interactions for single-molecule detection [54,55].

The enhancement factor in SERS can also be improved by coupling plasmon resonances with whispering-gallery resonances. Y. Mei and coworkers prepared a specific nanofluidic channel created by a strain-engineered self-rolling process [56]. Rolling and self-assembly of Ag NPs were achieved by a thermal annealing process that releases a strained SiO/TiO_2_/Ag thin films from a sacrificial polymer layer to form a rolled-up nanotube while simultaneously triggering the self-assembly of the Ag NPs, as described in Figure 2c. The sample was introduced into the nanocavity by capillary force. The cavity supports the coupling of plasmon and whispering-gallery resonances, which results in an additional enhancement of Raman scattering signals on the order of 10^5^, compared to that achieved by non-resonant flat SERS substrates.

##### Surface-Enhanced Infrared Absorption Spectroscopy (SEIRA)

Similar to SERS, SEIRA involves the coupling of photon and vibrational modes of molecules when they are located in the hot-spots of a propagating or localized surface plasmon polariton. The emergence of metamaterials, which are artificial materials composed of metallodielectric nanostructures, has allowed a new degree of freedom to engineer hot-spots in order to improve the enhancement factor in SEIRA. Many micro/nanofluidic devices integrated with metamaterials were demonstrated for in-situ probing of biomolecule interactions through SEIRA [57,58,59]. SEIRA provides spectroscopic information complementary to SERS, and its signals are usually stronger than those in SERS; however, the difficulty in fabricating IR-compatible device limits its application in micro/nanofluidics. For IR measurement, the device should transmit IR light, or at least possess an IR transparent window. In addition, the device should achieve a good transmission signal of the probed species regardless of the solvent absorption, especially water. Thus, instead of commonly used substrate materials such as SiO_2_ or PDMS, materials transparent to IR light, including calcium fluoride (CaF_2_), sapphire, zinc selenide, or silicon should be considered. In such a case, issues related to sealing and bonding still exist.

**Figure 3:**
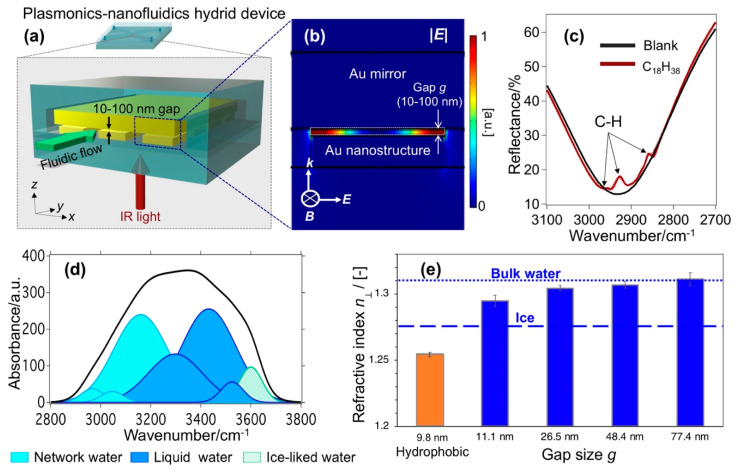
(**a**) Plasmonics-nanofluidics hybrid device consisting of an Au mirror and an array of periodic Au nanostructures separated by a nanofluidic channel, (**b**) the numerical calculation result of electric field profile *|**E**|* indicating the accumulation of light energy inside the nanogap as hot-spots, (**c**) vibrational modes of target molecules detected as peaks in the reflectance dip of the original plasmon resonance (adapted with permission from [60]. Copyright 2017 American Chemical Society), (**d**) vibrational absorption of water confined in a 10 nm gap (adapted with permission from [61]. Copyright 2018 American Chemical Society), and (**e**) Out-of-plane refractive index $n_{⊥}$ of water confined in 10–100 nm gaps (adapted with permission from [62]. Copyright 2020 The Royal Society of Chemistry).

By developing a robust bonding technique for SiO_2_–SiO_2_ or SiO_2_–CaF_2_ at room temperature, Le et al. realized a sophisticated nanofluidic device integrated with metamaterials (i.e., plasmonics–nanofluidics hybrid device) and used it for conducting SEIRA [60]. As shown in Figure 3a, the device comprises an Au mirror and an array of periodic Au nanostructures separated by a nanofluidic channel. The nanogap *g* between the Au nanostructures and Au mirror is controlled at several tens of nanometers (10–100 nm). The device exhibits a strong plasmon resonant mode in the mid-IR regime, which originates from the antiparallel currents excited on Au nanostructure and Au mirror, forming a quadrupole mode. This quadrupole mode is non-radiative one, thus very small amount of light is reflected back. Since the mirror layer blocks all the transmitted light, the combination of nearly-zero reflectance (*R*) and transmittance (*T*) results in a perfect absorption of light. This structure is well-known as metal-insulator-metal (MIM) or perfect absorber metamaterial. The numerical calculation reveals that at resonance, hot-spots accumulate within the nanogaps, as shown in electric field profile in Figure 3b. This unique configuration enables a “flow-through” apparatus, in which target analytes are introduced into the hot-spots of metamaterials by fluidic operation. It is in contrast with the “adsorb-on” scheme in previous reports using MIM metamaterial, where target molecules were adsorbed on-top of MIM structures. When molecules whose vibrational absorption overlaps with the resonant mode of the plasmonic structure are introduced into the gap, their vibrational modes couple with this plasmon resonance, resulting in the destruction of the above-mentioned quadrupole mode. The molecular vibrational modes are consequently observed as peaks in the reflectance dip of the original plasmon mode. For example, Figure 3c shows the spectrum of device when it is filled with C_18_H_38_ solution (in CCl_4_), and three stretching modes of C–H are clearly observed. This device recorded a sensitivity enhancement of up to two orders of magnitude compared to the state-of-the-art MIM metamaterial-based SEIRA. This result verified that the controllable delivery of molecules into hot-spots facilitated the plasmonic–molecular coupling, and thus increased the sensitivity. This device not only considerably improves sensitivity but also addresses some critical issues in SEIRA including those related to quantitative measurement and measurement in absorptive solvents such as water.

The spatial localization of plasmon fields within the gap as explained in Figure 3b suggests that it is promising to use this device to characterize the molecular structures of water confined in the nanogap. Le et al. has modified the device to measure the IR absorption spectra and elucidated the molecular structures of water confined in 10–100 nm gap [61]. The device was purposefully designed so that its plasmon resonance spectrally overlapped with the O–H stretching band of H_2_O at 3000–3600 cm^−1^. The absorption spectrum of water confined in a ~10 nm gap shown in Figure 3d reveals a large ratio of the low-wavenumber components (∼3200 cm^−1^) (i.e., network water) with respect to the component at ∼3400 cm^−1^ (i.e., liquid-water), indicating the presence of a stronger H-bond network in nanoconfined water compared to that in bulk water. They also tuned the sizes and the interfacial properties of nanogaps by physical and chemical modifications to further elucidate this effect. The results revealed subtle differences in the spectra with decreasing gap sizes, revealing the scaling behavior of confined water in 10–100 nm gaps. It should be noticed from their results that these properties are not impacted by the interactions with the interfaces, but the constrained geometry itself promotes the intermolecular interactions of water and strengthens the H-bond network.

Further exploiting the unique characteristics of the hot-spots in their devices, they also measured the out-of-plane refractive index ($n_{⊥}$; i.e., the direction perpendicular to the confining walls) of water confined in 10–100 nm gaps, as the plasmon resonant wavelength shifts [62]. The results in Figure 3e clearly show a statistically significant difference in $n_{⊥}$ values in devices of different gap sizes and interfacial properties. In hydrophilic gaps, $n_{⊥}$ gradually decreases with small gap *g*; it reaches an intermediate value between that of bulk water and hexagonal ice in a gap of ~10 nm, while it recovers the bulk value in a gap of close to 80 nm. In a 10 nm cavity of hydrophobic interfaces, RI exhibits an anomalously low value. Not only is it much lower than that of hydrophilic interfaces of the same gap, but even lower than that of hexagonal ice. Employing RI as a sensing probe, they also observed the anomalous stability of water structures over a wide range of temperature, which is also explained by the strengthened H-bonds. These plasmonics–nanofluidics hybrid devices pave a new methodology of using plasmon resonance to characterize nanoconfined molecules and nanoconfined chemical reactions, and thus provide fundamental insight into the confinement effects.

### 2.4. Photothermal Detection

Photothermal detection is one of the methods to sensitively measure the absorbance of an analyte. The detection method was originally reported as thermal lens calorimetry [63] and developed as thermal lens microscopy for microfluidic chips [64]. The principle of photothermal detection is based on optical absorption and subsequent nonradiative (thermal) relaxation process of analyte molecules. The photothermal conversion of energy is mainly detected through a change in refractive index of the medium. Refractive indices of most liquids decrease with rising temperature. Therefore, when an excitation beam, which is absorbed by the analyte molecules, is a focused Gaussian beam, the distribution of refractive index is equivalent to a concave lens. This is called as a thermal lens effect and refraction or deflection by the thermal lens is detected by using a probe beam.

**Figure 4:**
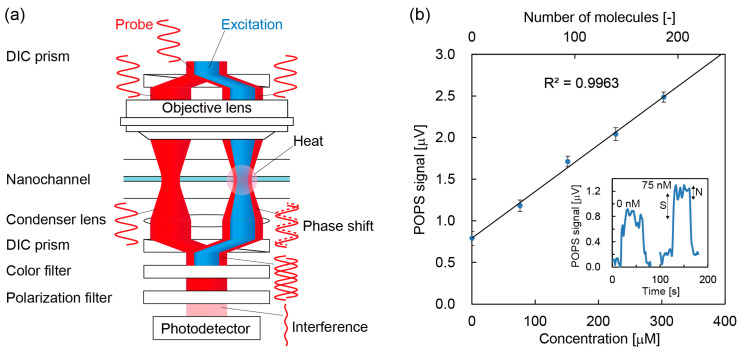
(**a**) Principle of photothermal optical phase shift (POPS). A probe beam (red) is separated by a differential interference contrast (DIC) prism and integrated by another DIC prism. An excitation beam (blue) is not separated and induces a photothermal effect (heat followed by a change in refractive index). The photothermal effect produces a phase shift for one of the probe beams and the phase shift is detected through an interference. (**b**) Detection of non-labeled bovine serum albumin by POPS. The limit of detection was 50 nM (30 molecules) (adapted with permission from [66]. Copyright 2020 The Royal Society of Chemistry).

Nevertheless, the principle based on refraction by the thermal lens was difficult to apply to nanochannels because the thermal lens induced in the nanochannel is smaller than the wavelength of light. Hence, a novel principle which is different from geometrical optics such as refraction and deflection is required. Photothermal detection in nanochannels was first realized by introducing the principle of DIC by Shimizu et al. [65]. In the principle of photothermal optical phase shift (POPS) detection, as shown in Figure 4a, a probe beam is separated into two beams and interferes in the detection of the photothermal phase contrast induced by an excitation beam. Here, the intensity of the probe beam is zero when no photothermal effect is induced in the nanochannel, which enables background-free detection. Moreover, the principle is based on interference which can be effective in a nanospace smaller than the wavelength. Thus, both background-free detection and wave optics are significant advantages in detection in nanochannel. The targets of POPS detection are all molecules which have optical absorption in UV/vis range. For example, ~30 protein molecules were detected by the UV-POPS detector as shown in Figure 3b [66].

However, the dissipation of thermal energy from the liquid sample to the nanochannel wall decreases the photothermal sensitivity in nanochannels. In some cases, the cancellation of the changes in refractive indices becomes another problem because the temperature-gradients of refractive indices (dn/dT) of water and silica are −9.1 × 10^−5^ and 9.8 × 10^−6^ K^−1^, respectively (at 20 °C). Therefore, the sensitive measurement using POPS in less than 500 nm deep channel was difficult. Le et al. tried to solve these problems by modifying the nanochannel wall with TiO_2_ which have a negative dn/dT [67]. Because the dn/dT of TiO_2_ in the rutile phase is −3.0 to −1.8 × 10^−4^ K^−1^, the temperature increase of TiO_2_ enhances the POPS signal. The enhancement of the POPS signal in 50 nm deep TiO_2_ channel was considerably effective, while the POPS signal was not detected in the SiO_2_ channel with the same depth. Another approach to the smaller nanochannel using photothermal optical diffraction (POD) was conducted by Tsuyama et al. [68]. In the POD experiment, the probe beam was irradiated to a single nanochannel to observe a diffracted light. Here, the intensity of the diffracted light depends on the difference of refractive indices of the sample solution and silica. Next, when the excitation beam was irradiated to the same nanochannel to induce the photothermal effect, the difference of refractive indices increases because the dn/dT of water and silica are negative and positive, respectively. Then, the intensity change of the diffracted light is observed as a POD signal. The POD detection showed higher sensitivities for ~300 nm deep channels.

As well as scattering-based and plasmonic detection, the use of hollow-core optical fiber is an effective approach for photothermal spectroscopy to deliver and couple light and molecules. Although liquid-phase photothermal measurements were not implemented in hollow-core optical fibers as far as the authors know, Zhao et al. reported ultrasensitive gas sensing based on the mode-phase difference [69].

## 3. Electrical/Electrochemical Detection

Electrical detection methods are also effective for label-free detection. However, considering the small size of a nanochannel, electrical detection will be difficult because of the large impedance of the liquid in the nanochannel (GΩ~TΩ). In addition, extremely small current signals (at the picoampere-to-femtoampere scale) will be required to detect molecules with countable molecule levels confined in nanochannels. In this section, the detection of the liquid itself in the nanochannel and the detection of molecules with countable molecule levels were presented.

### 3.1. Detection Based on Conductivity Measurement

The most famous method for conductivity-based detection is resistive pulse sensing using nanopores. In this method, conductivity change when a molecule flow through the nanopore is detected by a change of electric current. Regarding this method, numerous papers were reported and summarized in the previous reviews in these 15 years [70,71,72,73,74,75,76]. In this section, some prominent works are selected from the recent reports and introduced briefly. Waduge et al. detected a protein using a nanopore with a diameter of 4.5 nm and discriminated different structures of a protein due to its conformational change [77]. Lin et al. detected a lysozyme using a nanopore with a diameter of 21 ± 4 nm [78]. In this report, the conductivity change of DNA aptamer-functionalized Au nanoparticles (NPs) due to binding of lysozymes was detected. Ohshiro et al. performed single DNA sequencing using gold electrodes which have a 0.75 nm gap [79]. In this report, sequencing of DNAs including two types of base sequences was realized by conductance measurements of a single DNA molecule and their abundance ratio in the solution was determined by counting the number of the molecules. Heerema et al. detected translocation of a DNA using a nanopore with a diameter of 5 nm located in the center of a 30 nm × 30 nm graphene nanoribbon [80]. In this report, the nanopore on the graphene nanoribbon allowed a measurement of resistive modulations using the in-plane current due to the DNA translocation. Giamblanco et al. detected aggregated proteins using a nanopore with a diameter of 20.7 ± 0.7 nm [81]. In this report, the current signal was detected when the aggregated proteins transited the nanopore with preventing aggregation on the nanopore surface and nanopore clogging.

**Figure 5:**
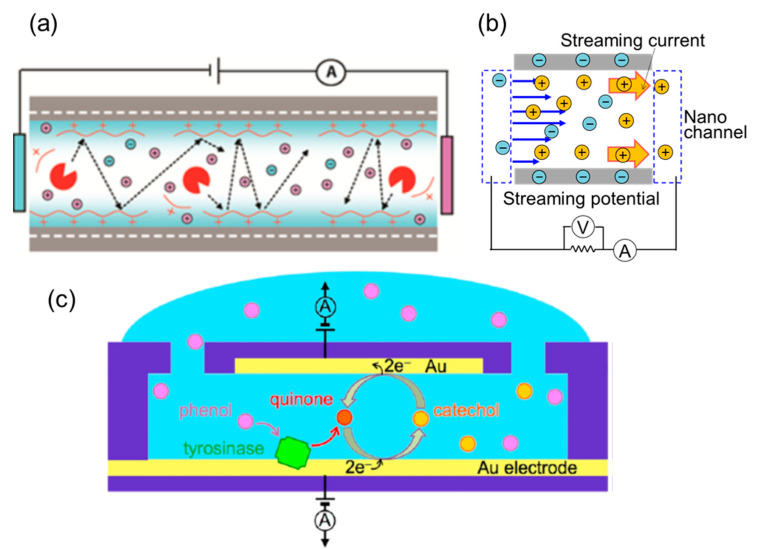
Nanofluidic devices for electrical/electrochemical detection, (**a**) detection of conductivity on nanochannel surfaces during chemical reaction (adapted with permission from [90]. Copyright 2016 American Chemical Society), (**b**) detection of streaming current/potential induced by pressure-driven flow in a nanochannel (adapted with permission from [115]. Copyright 2015 American Chemical Society), and (**c**) detection of electrochemical reaction using electrodes integrated in a nanochannel. (adapted with permission from [120]. Copyright 2014 American Chemical Society).

As well as the nanopores, the development of the fabrication technologies of nanochannels also realized conductivity-based sensing in a nanochannel. Utilizing top-down fabrication techniques such as lithography, sputtering, etching, and bonding, complicated channel shapes were fabricated and even nanoelectrodes were integrated into the nanochannels. The electrical conductivity of liquid in nanochannels (width: 50 μm and depth: 70, 180, 380, 590, and 1015 nm) was measured for the first time by Stein et al. [82]. The conductivity of liquid in nanogaps (width: 56–815 nm and depth: 100 nm) and nanopores (diameter: 140, 160, 250, 430, and 520 nm) were also measured by other groups [83,84]. In their results, the measured conductivity corresponded to the conductivity of bulk liquid in the mM concentration area; however, in the μM concentration area, the effect of the surface charge on the nanochannel was revealed. In addition, in such a surface-governed space, the effect of the electric double layer (EDL) near the nanochannel surface on the nanoscale space is not negligible. The thickness of the EDL is given by a Debye length which depends on the ionic strength of the liquid phase. The Debye length for the mM concentration is 1–10 nm, while the Debye length for the μM concentration is 10–300 nm. In the μM concentration area in their experimental conditions, the thickness of EDL exceeded the channel/pore size, which is called an EDL overlap. Because conductivity of the EDL is usually larger than that of the bulk liquid, the conductivities measured in the nanochannels and nanopores were larger than that of bulk liquid. Using the surface-governed property, surface sensors for the nanochannels were developed. For example, Karnik et al. (width: 10 μm and depth: 30 nm) and Schoch et al. (width: 5.5 μm and depth: 50 nm) measured the conductivity change due to the biotin–streptavidin reaction on the nanochannel surfaces [85,86]. Using the same principle, Durand et al. detected protein adsorption on the nanochannel surface [87]. They measured the conductivity change in the nanochannel (width: 2.5 mm and depth: 50 nm) due to the adsorption of bovine serum albumin. Hsueh et al. detected a biomarker with the range from 100 pg/mL to 10 μg/mL using a nanogap [88]. They measured the capacitance change of the nanogap (width: 200 nm and depth: 20 nm) due to the binding of cardiac troponin T (cTnT) on the surface. Liao et al. detected a microRNA with the range from 1 fM to 1 nM on the nanochannel surface [89]. Using a conical nanochannel which has diameters of 1.1 μm and 51 nm, the conductivity change due to hybridization of microRNAs on the nanochannel surface was measured. Duan et al. detected an enzyme reaction on the nanochannel surface as shown in Figure 5a [90]. They used a nanochannel (width: 2 μm and depth: 52 nm) and measured the conductivity change owing to enzymatic reaction by Trypsin with the range from 5 ng/mL to 50 μg/mL. For applications using an overlapped electric double layer in nanochannels, ion concentration effects induced by nanochannels that functioned as ion-selective channels were reported. Kim et al. (depth: 40 nm) [91], Chang et al. (width: 100 μm and depth: 60 nm) [92], Huang et al. (width: 100 μm and depth: 100 nm) [93], and Ahmed et al. (diameter: 70 nm) [94] determined the concentration of typical ions. Furthermore, expanding the principle of the ion concentration, ion rectification devices were developed by combining patterning of different surface charges in a nanochannel (width: 4 μm and depth: 30 nm) [95], nanopore (diameter: 10–30 nm at the small opening and 250–300 nm at the large opening) [96], or nanofunnel (width: 80 nm and depth: 120 nm at the small opening and width: 1 μm and depth: 120 nm at the large opening) [97]. Applying in-situ voltage to electrodes fabricated in a nanochannel (width: 22 and 33 μm and depth: 20 nm), Guan et al. also developed an ionic diode device [98]. Eberle et al. controlled the macromolecular motion in a nanochannel (width: 500 nm and depth: 200 nm) by the valve of electric potentials [99]. Another approach to detect conductivity changes is to vary the micro/nanochannel interface shape, whereas Lin et al. detected differences in electrical resistance before and after connecting a living single cell and a nanochannel (width: 900 nm and depth: 900 nm) by lipid fusion which is well known as patch clump method [100,101]. The number of molecules were detected by determining the changes in conductivity as the analyte passes through the nanochannel. Peng et al. (width: 1.1 μm and depth: 920 nm) detected DNA [102], Harms et al. (width: 50 nm and depth: 50 nm) [103] and Kondylis et al. (width: 100 nm and depth: 100 nm) [104] detected viruses in nanochannels, and Yasaki et al. (width: 2.2 μm and depth: 3.7 μm) detected bacterial cells in a microchannel [105]. In their methods, advanced analyses were achieved by integrating the pore structures in micro/nanochannels which were fabricated by top-down methods. These approaches may accelerate the integration of detection based on resistive pulse sensing with other analytical procedures such as pretreatment and separation in an advanced micro/nanochannel network.

### 3.2. Detection Based on Electrokinetic Phenomena

In a charged nanochannel, an EDL is formed near the surface. When an electric potential is applied to the nanochannel, liquid flow is induced because of an osmosis of ions. This flow is called electro-osmotic flow (EOF) and is mainly detected by monitoring fluorescent probes [106,107]. In the recent reports by Li’s group (width: 183 ± 11 nm and depth: 42 ± 6 nm), EOF was detected by monitoring current, which is considered as a label-free method [108]. They also detected EOF in a DNA-modified nanochannel (width: 87 nm and depth: 50 nm, width: 265 nm and depth: 104 nm, width: 865 nm and depth: 650 nm, and width: 2.5 μm and depth: 2.5 μm, respectively) [109]. In contrast to EOF, when a pressure is applied to liquid in a nanochannel, the pressure-driven flow of ions induces an electric potential/current; this is called a streaming potential/current. In this method, electric signals depend on the flow rate of the liquid and zeta potential of the nanochannel surface. van der Heyden et al. measured the streaming potential/current in a nanochannel (width: 50 μm and depth: 70 nm, 140 nm, 279 nm, 563 nm, and 1047 nm) and the charge density of the nanochannel surface and energy conversion efficiency were determined [110,111,112]. Morikawa et al. measured the streaming potentials/currents in square nanochannels (width: 380 nm and depth: 350 nm, width: 610 nm and depth: 560 nm, width: 1200 nm and depth: 950 nm, and width: 1580 nm and depth: 1730 nm, respectively) [113]. The detected steaming current signal was pico-ampere order due to the extremely low flow rate (pico-liter per minute order) in the square nanochannels. In addition, they reported unique liquid properties in the nanospaces such as enhanced disassociation of chemical surface groups and lower dielectric constants compared to bulk liquid [114,115]. Xu et al. also detected streaming currents in a nanochannel (width: 724 nm and depth: 292 nm) using nano-electrodes integrated in a nanofluidic device [116]. Siria et al. measured the streaming and osmotic currents in single nanotubes (diameter: 15 nm, 22 nm, 29 nm, 49 nm) and discussed the osmotic energy conversions [117]. In this way, liquid motion and its properties can be detected using electrokinetic phenomena.

### 3.3. Electrochemical Detection

One of the major issues in detecting electrochemical reactions in nanochannels is the integration of electrodes into the nanochannels. Particularly, the precise fabrication of the nanochannel and nano-electrode is required as well as their precise arrangement. Lemay’s group detected redox-active molecules at pM level in a nanochannel (width: 1.5 μm and depth: 70 nm) by measuring femtoampere-scale current signals [118,119]. Their method was also applied to a bionanofluidic sensor using a nanochannel (width: 3 μm and depth: 200 nm) in a different reaction scheme [120]. In addition, the time of flight between two electrochemical reactions in each electrode was measured, and their method further applied to a flow meter with picoliter-per-minute level in a nanochannel (width: 5 μm and depth: 130 nm) [121]. Among other groups, Sanghavi et al. performed immunoassays in the nanochannel (width: 30 μm and depth: 200 nm) and determined the electrochemical reaction [122]. Although reports of electrochemical detection in nanochannels are limited because of the challenging fabrication requirements, more effective label-free detection tools can be developed by improving the fabrication methods.

## 4. Outlook

Advances in nanofabrication and bonding techniques over the past decade have triggered the development of nanofluidics, and nanofluidic devices have been widely applied in various chemical and biological analyses. Detection techniques, particularly the label-free ones have contributed much to this growth, but they are still facing considerable challenges. Table 1 summarizes and categorizes all detection methods mentioned in this review in terms of target molecules and device configurations. In the next decade, analytical tools based on nanofluidics will surely advance in biochemical and biomedical research. Nanofluidic devices also provide a new platform for diagnosis and sensing that will be potentially commercialized and accepted by end users. Several research directions regarding label-free detections in nanofluidics can be envisioned; those could either facilitate the discovery of novel functionalities of nanofluidic analytical device or explore fundamental phenomena in nanofluidics.

### 4.1. Exploring Fundamental Phenomena in Nanofluidics

Many fundamental phenomena in nanofluidics, such as flow dynamics, slipping, and electrokinetic effects still need to be investigated [123]. The challenges to understand these phenomena include difficulty in imaging or visualizing fluidic flow at nanometer-scale spatial resolution. Several imaging techniques with excellent resolution using nanoparticles as tracers have been developed [124,125]; yet the observed results do not purely reflect the flow dynamics because of the large electrokinetic and entropic effects of nanoparticles. Developing new techniques such as imaging without tracers or conducting comprehensive studies using imaging and electrochemical methods are required to discover the dynamics of a nanofluidic flow. Despite extensive studies on distinctive properties of liquids confined in nanospaces, the underlying mechanism and the significance of the length-scale involved in the confinement effects are still subjects of controversy. In this light, spectroscopic methods are highly desired for in-situ probing the molecular structures and the kinetics of nanoconfined molecules or nanoconfined chemical reactions in their intact condition. The knowledge achieved will provide valuable insights into nanoconfinement effects, as well as promote the implementation of those effects into nanofluidic analytical devices.

### 4.2. Single Cell Analysis and Single-Cell Omics

Single-cell analysis, and single-cell omics in particular, increasingly gained attention due to the recognition of cell heterogeneity in cell biology [126,127]. Nanofluidic devices offer fascinating platforms to analyze complex biological systems at the single-cell level, because they allow the processing of sample volumes at the scale from attoliters to femtoliters, which is much smaller than the volume of a single cell (picoliters). In the near future, the integration of entire analytical processes including sample separation, purification, preconcentration, and multiple detection methods into a single nanofluidic device will enable a new single-cell molecular profiling system. Especially, a multimodal profiling platform is highly desired to build a comprehensive molecular perspective of the cell. Aside from detections, many techniques such as fluidic control, integration and functionalization of nanofluidic systems must be realized in order to achieve the goal of single-cell omics; nevertheless, nanofluidics would undoubtedly be an important direction in single-cell analysis.

### 4.3. Fully Integrated Diagnosis and Sensing Systems

The adaptation and integration of sample separation, purification, and preconcentration techniques such as of chromatography, electrophoresis, or photophoresis into a nanofluidic chip will significantly enhance the performance and broaden the capabilities of nanofluidics-based analytical devices [128]. Such automated and high-throughput analytical devices have the potential to replace conventional bulky, expensive, and manpower- and time-consuming ones. Furthermore, in the near future, the miniaturization of light sources, detectors, and signal read-out components may facilitate a comprehensive integration of entire analytical processes to provide a compact and portable analytical device for use in remote or resource-limited locations [129].

## Figures and Tables

**Table 1:** Summary of label-free detection methods applicable to nanofluidics.

		Targets		Liquid		Ion	Small Molecules		Large Molecules (DNA, Proteins, Peptides, etc.)	Nano-Particle	Virus	Bacteria
Methods												
**Optical**	Diffraction								[22,23,24]			
	Scattering									[27]	[28]	
	Plasmonics		RI	[37]			[40]		[38,39,40,42]			[39]
			SERS				[48,54,55]					
							[50,53]	[56]	[49,51,52]			
			SEIRA	[61,62]			[60]					
	Photothermal						[65,67,68]		[66]			
**Electrical**	Conductivity					[84,96]			[77,78,79,80,81,89]		[103,104]	[105]
						[82,83,91,92,93,94,95]			[85,86,87,88,90,99]			
						[97,98,100,101]			[102]			
	Electrokinetic			[108,109,110,111,112,113,114,115,116]	[117]							
	Electrochemical						[118,119,120,121]		[122]			

Structure: 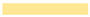
—Nanotube. 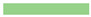
—Nanopore. 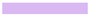
—Nanochannel.

## References

[1] Shang L., Cheng Y., Zhao Y. (2017). Emerging Droplet Microfluidics. Chem. Rev..

[2] Elvira K.S., Solvas X.C.I., Wootton R.C.R., de Mello A.J. (2013). The past, present and potential for microfluidic reactor technology in chemical synthesis. Nat. Chem..

[3] Zhang B., Korolj A., Lai B.F.L., Radisic M. (2018). Advances in organ-on-a-chip engineering. Nat. Rev. Mater..

[4] Morikawa K., Tsukahara T. (2014). Investigation of Unique Protonic and Hydrodynamic Behavior of Aqueous Solutions Confined in Extended Nanospaces. Isr. J. Chem..

[5] Schoch R.B., Han J., Renaud P. (2008). Transport phenomena in nanofluidics. Rev. Mod. Phys..

[6] Reisner W., Morton K.J., Riehn R., Wang Y.M., Yu Z., Rosen M., Sturm J.C., Chou S.Y., Frey E., Austin R.H. (2005). Statics and dynamics of single DNA molecules confined in nanochannels. Phys. Rev. Lett..

[7] Reisner W., Larsen N.B., Silahtaroglu A., Kristensen A., Tommerup N., Tegenfeldt J.O., Flyvbjerg H. (2010). Single-molecule denaturation mapping of DNA in nanofluidic channels. Proc. Natl. Acad. Sci. USA.

[8] Lam E.T., Hastie A., Lin C., Ehrlich D., Das S.K., Austin M.D., Deshpande P., Cao H., Nagarajan N., Xiao M. (2012). Genome mapping on nanochannel arrays for structural variation analysis and sequence assembly. Nat. Biotechnol..

[9] Cipriany B.R., Zhao R., Murphy P.J., Levy S.L., Tan C.P., Craighead H.G., Soloway P.D. (2010). Single Molecule Epigenetic Analysis in a Nanofluidic Channel. Anal. Chem..

[10] Han J., Craighead H.G. (2000). Separation of long DNA molecules in a microfabricated entropic trap array. Science.

[11] Kaji N., Tezuka Y., Takamura Y., Ueda M., Nishimoto T., Nakanishi H., Horiike Y., Baba Y. (2004). Separation of long DNA molecules by quartz nanopillar chips under a direct current electric field. Anal. Chem..

[12] Ishibashi R., Mawatari K., Kitamori T. (2012). Highly efficient and ultra-small volume separation by pressure-driven liquid chromatography in extended nanochannels. Small.

[13] Wang Y.C., Stevens A.L., Han J.Y. (2005). Million-fold preconcentration of proteins and peptides by nanofluidic filter. Anal. Chem..

[14] Fontana M., Fijen C., Lemay S.G., Mathwig K., Hohlbein J. (2019). High-throughput, non-equilibrium studies of single biomolecules using glass-made nanofluidic devices. Lab Chip.

[15] Tsukahara T., Mawatari K., Kitamori T. (2010). Integrated extended-nano chemical systems on a chip. Chem. Soc. Rev..

[16] Segerink L.I., Eijkel J.C.T. (2014). Nanofluidics in point of care applications. Lab Chip.

[17] Abgrall P., Nguyen N.T. (2008). Nanofluidic Devices and Their Applications. Anal. Chem..

[18] Piruska A., Gong M., Sweedler J.V., Bohn P.W. (2010). Nanofluidics in chemical analysis. Chem. Soc. Rev..

[19] Zhou K., Perry J.M., Jacobson S.C. (2011). Transport and Sensing in Nanofluidic Devices. Annu. Rev. Anal. Chem..

[20] Yu H., Peng Y., Yang Y., Li Z. (2019). Plasmon-enhanced light–matter interactions and applications. NPJ Comput. Mater..

[21] Dumond J.J., Low H.Y., Rodriguez I. (2006). Isolated, sealed nanofluidic channels formed by combinatorial-mould nanoimprint lithography. Nanotechnology.

[22] Yasui T., Ogawa K., Kaji N., Nilsson M., Ajiri T., Tokeshi M., Horiike Y., Baba Y. (2016). Label-free detection of real-time DNA amplification using a nanofluidic diffraction grating. Sci. Rep..

[23] Purr F., Bassu M., Lowe R.D., Thürmann B., Dietzel A., Burg T.P. (2017). Asymmetric nanofluidic grating detector for differential refractive index measurement and biosensing. Lab Chip.

[24] Purr F., Eckardt M., Kieserling J., Gronwald P., Burg T.P., Dietzel A. (2019). Robust smartphone assisted biosensing based on asymmetric nanofluidic grating interferometry. Sensors.

[25] Enoki S., Iino R., Morone N., Kaihatsu K., Sakakihara S., Kato N., Noji H. (2012). Label-free single-particle imaging of the influenza virus by objective-type total internal reflection dark-field microscopy. PLoS ONE.

[26] Piliarik M., Sandoghdar V. (2014). Direct optical sensing of single unlabelled proteins and super-resolution imaging of their binding sites. Nat. Commun..

[27] Mitra A., Deutsch B., Ignatovich F., Dykes C., Novotny L. (2010). Nano-optofluidic detection of single viruses and nanoparticles. ACS Nano.

[28] Faez S., Lahini Y., Weidlich S., Garmann R.F., Wondraczek K., Zeisberger M., Schmidt M.A., Orrit M., Manoharan V.N. (2015). Fast, label-free tracking of single viruses and weakly scattering nanoparticles in a nanofluidic optical fiber. ACS Nano.

[29] Fan X., White I.M. (2011). Optofluidic microsystems for chemical and biological analysis. Nat. Photon..

[30] Psaltis D., Quake S.R., Yang C. (2006). Developing optofluidic technology through the fusion of microfluidics and optics. Nature.

[31] Monat C., Domachuk P., Eggleton B.J. (2007). Integrated optofluidics: A new river of light. Nat. Photon..

[32] Fainman Y., Psaltis D., Yang C. (2010). Optofluidics: Fundamentals, Devices, and Applications.

[33] Testa G., Huang Y., Sarro P.M., Zeni L., Bernini R. (2010). Integrated optofluidic ring resonator. Appl. Phys. Lett..

[34] Shopova S.I., White I.M., Sun Y., Zhu H., Fan X., Frye-Mason G., Thompson A., Ja S. (2008). On-column micro gas chromatography detection with capillary-based optical ring resonators. Anal. Chem..

[35] Lin S., Schonbrun E., Crozier K. (2010). Optical manipulation with planar silicon microring resonators. Nano Lett..

[36] Soltani M., Lin J., Forties R.A., Inman J.T., Saraf S.N., Fulbright R.M., Lipson M., Wang M.D. (2014). Nanophotonic trapping for precise manipulation of biomolecular arrays. Nat. Nanotechnol..

[37] Huang M., Yanik A.A., Chang T., Altug H. (2009). Sub-wavelength nanofluidics in photonic crystal sensors. Opt. Express.

[38] Eftekhari F., Escobedo C., Ferreira J., Duan X., Girotto E.M., Brolo A.G., Gordon R., Sinton D. (2009). Nanoholes as nanochannels: Flow-through plasmonic sensing. Anal. Chem..

[39] Gomez-Cruz J., Nair S., Manjarrez-Hernandez A., Gavilanes-Parra S., Ascanio G., Escobedo C. (2018). Cost-effective flow-through nanohole array-based biosensing platform for the label-free detection of uropathogenic E. coli in real time. Biosens. Bioelectron..

[40] Escobedo C., Brolo A.G., Gordon R., Sinton D. (2010). Flow-through vs flow-over: Analysis of transport and binding in nanohole array plasmonic biosensors. Anal. Chem..

[41] Nakao T., Kazoe Y., Morikawa K., Lin L., Mawatari K., Kitamori T. (2020). Femtoliter volumetric pipette and flask utilizing nanofluidics. Analyst.

[42] Guo Y., Li H., Reddy K., Shelar H., Nittoor V., Fan X. (2011). Optofluidic Fabry-Pérot cavity biosensor with integrated flow-through micro-/nanochannels. Appl. Phys. Lett..

[43] Artar A., Yanik A., Altug H. (2009). Fabry- Pérot nanocavities in multilayered plasmonic crystals for enhanced biosensing. Appl. Phys. Lett..

[44] Aroca R. (2006). Surface Enhanced Vibrational Spectroscopy.

[45] Kneipp K., Wang Y., Kneipp H., Perelman L.T., Itzkan I., Dasari R.R., Feld M.S. (1997). Single molecule detection using surface-enhanced raman scattering (SERS). Phys. Rev. Lett..

[46] Adato R., Altug H. (2013). In-situ ultra-sensitive infrared absorption spectroscopy of biomolecule interactions in real time with plasmonic nanoantennas. Nat. Commun..

[47] Dong L., Yang X., Zhang C., Cerjan B., Zhou L., Tseng M.L., Zhang Y., Alabastri A., Nordlander P., Halas N.J. (2017). Nanogapped Au antennas for ultrasensitive surface-enhanced infrared absorption spectroscopy. Nano Lett..

[48] Kumar S., Cherukulappurath S., Johnson T.W., Oh S. (2014). Millimeter-sized suspended plasmonic nanohole arrays for surface-tension-driven flow-through SERS. Chem. Mater..

[49] Chen R., Du X., Cui Y., Zhang X., Ge Q., Dong J., Zhao X. (2020). Vertical flow assay for inflammatory biomarkers based on nanofluidic channel array and SERS nanotags. Small.

[50] Zhao X., Xue J., Mu Z., Huang Y., Lu M., Gu Z. (2015). Gold nanoparticle incorporated inverse opal photonic crystal capillaries for optofluidic surface enhanced Raman spectroscopy. Biosens. Bioelectron..

[51] Chou I., Benford M., Beier H.T., Coté G.L., Wang M., Jing N., Kameoka J., Good T.A. (2008). Nanofluidic biosensing for β-amyloid detection using surface enhanced raman spectroscopy. Nano Lett..

[52] Choi I., Huh Y.S., Erickson D. (2011). Size-selective concentration and label-free characterization of protein aggregates using a Raman active nanofluidic device. Lab Chip.

[53] Takeshita T., Suekuni K., Aiba K., Sugano K., Isono Y. (2017). Surface-enhanced Raman spectroscopy analysis device with gold nanoparticle arranged nanochannel. Electron. Commun. Jpn..

[54] Chen C., Li Y., Kerman S., Neutens P., Willems K., Cornelissen S., Lagae L., Stakenborg T., Dorpe P.V. (2018). High spatial resolution nanoslit SERS for single-molecule nucleobase sensing. Nat. Commun..

[55] Huang J., Mousavi M.Z., Zhao Y., Hubarevich A., Omeis F., Giovannini G., Schütte M., Garoli D., Angelis F.D. (2019). SERS discrimination of single DNA bases in single oligonucleotides by electro-plasmonic trapping. Nat. Commun..

[56] Zhang J., Li J., Tang S., Fang Y., Wang J., Huang G., Liu R., Zheng L., Cui X., Mei Y. (2015). Whispering-gallery nanocavity plasmon-enhanced Raman spectroscopy. Sci. Rep..

[57] Yoo D., Mohr D.A., Vidal-Codina F., John-Herpin A., Jo M., Kim S., Matson J., Caldwell J.D., Jeon H., Nguyen N. (2018). High-Contrast Infrared Absorption Spectroscopy via Mass-Produced Coaxial Zero-Mode Resonators with Sub-10 nm Gaps. Nano Lett..

[58] Rodrigo D., Tittl A., Ait-Bouziad N., John-Herpin A., Limaj O., Kelly C., Yoo D., Wittenberg N.J., Oh S., Lashuel H.A. (2018). Resolving molecule-specific information in dynamic lipid membrane processes with multi-resonant infrared metasurfaces. Nat. Commun..

[59] Bomers M., Charlot B., Barho F., Chanuel A., Mezy A., Cerutti L., Gonzalez-Posada F., Taliercio T. (2020). Microfluidic surface-enhanced infrared spectroscopy with semiconductor plasmonics for the fingerprint region. React. Chem. Eng..

[60] Le H.H.T., Tanaka T. (2017). Plasmonics-Nanofluidics Hydrid Metamaterial: An Ultrasensitive Platform for Infrared Absorption Spectroscopy and Quantitative Measurement of Molecules. ACS Nano.

[61] Le H.H.T., Morita A., Mawatari K., Kitamori T., Tanaka T. (2018). Metamaterials-Enhanced Infrared Spectroscopic Study of Nanoconfined Molecules by Plasmonics-Nanofluidics Hydrid Device. ACS Photonics.

[62] Le H.H.T., Morita A., Tanaka T. (2020). Refractive index of nanoconfined water reveals its anomalous physical properties. Nanoscale Horiz..

[63] Dovichi N.J., Harris J.M. (1979). Laser induced thermal lens effect for calorimetric trace analysis. Anal. Chem..

[64] Tokeshi M., Uchida M., Hibara A., Sawada T., Kitamori T. (2001). Determination of subyoctomole amounts of nonfluorescent molecules using a thermal lens microscope:  subsingle-molecule determination. Anal. Chem..

[65] Shimizu H., Mawatari K., Kitamori T. (2010). Sensitive determination of concentration of nonfluorescent species in an extended-nano channel by differential interference contrast thermal lens microscope. Anal. Chem..

[66] Shimizu H., Takeda S., Mawatari K., Kitamori T. (2020). Ultrasensitive detection of nonlabelled bovine serum albumin using photothermal optical phase shift detection with UV excitation. Analyst.

[67] Le H.H.T., Mawatari K., Shimizu H., Kitamori T. (2014). Detection of zeptomole quantities of nonfluorescent molecules in a 10^1^ nm nanochannel by thermal lens microscopy. Analyst.

[68] Tsuyama Y., Mawatari K. (2019). Nonfluorescent molecule detection in 10^2^ nm nanofluidic channels by photothermal optical diffraction. Anal. Chem..

[69] Zhao P., Zhao Y., Bao H., Ho H.L., Jin W., Fan S., Gao S., Wang Y., Wang P. (2020). Mode-phase-difference photothermal spectroscopy for gas detection with an anti-resonant hollow-core optical fiber. Nat. Commun..

[70] Dekker C. (2007). Solid-state nanopores. Nat. Nanotechnol..

[71] Howorka S., Siwy Z. (2009). Nanopore analytics: Sensing of single molecules. Chem. Soc. Rev..

[72] Siwy Z.S., Howorka S. (2010). Engineered voltage-responsive nanopores. Chem. Soc. Rev..

[73] Mulero R., Prabhu A.S., Freedman K.J., Kim M.J. (2010). Nanopore-Based Devices for Bioanalytical Applications. J. Assoc. Lab. Autom..

[74] Hou X., Guo W., Jiang L. (2011). Biomimetic smart nanopores and nanochannels. Chem. Soc. Rev..

[75] Miles B.N., Ivanov A.P., Wilson K.A., Dogan F., Japrung D., Edel J.B. (2013). Single molecule sensing with solid-state nanopores: Novel materials, methods, and applications. Chem. Soc. Rev..

[76] Shi W., Friedman A.K., Baker L.A. (2017). Nanopore Sensing. Anal. Chem..

[77] Waduge P., Hu R., Bandarkar P., Yamazaki H., Cressiot B., Zhao Q., Whitford P.C., Wanunu M. (2017). Nanopore-Based Measurements of Protein Size, Fluctuations, and Conformational Changes. ACS Nano.

[78] Lin X., Ivanov A.P., Edel J.B. (2017). Selective single molecule nanopore sensing of proteins using DNA aptamer-functionalised gold nanoparticles. Chem. Sci..

[79] Ohshiro T., Tsutsui M., Yokota K., Taniguchi M. (2018). Quantitative analysis of DNA with single-molecule sequencing. Sci. Rep..

[80] Heerema S.J., Vicarelli L., Pud S., Schouten R.N., Zandbergen H.W., Dekker C. (2018). Probing DNA Translocations with Inplane Current Signals in a Graphene Nanoribbon with a Nanopore. ACS Nano.

[81] Giamblanco N., Coglitore D., Janot J.M., Coulon P.E., Charlot B., Balme S. (2018). Detection of protein aggregate morphology through single antifouling nanopore. Sens. Actuat. B Chem..

[82] Stein D., Kruithof M., Dekker C. (2004). Surface-Charge-Governed Ion Transport in Nanofluidic Channels. Phys. Rev. Lett..

[83] Hatsuki R., Yujiro F., Yamamoto T. (2013). Direct measurement of electric double layer in a nanochannel by electrical impedance spectroscopy. Microfluid. Nanofluid..

[84] Lee C., Joly L., Siria A., Biance A., Fulcrand R., Bocquet L. (2012). Large Apparent Electric Size of Solid-State Nanopores Due to Spatially Extended Surface Conduction. Nano Lett..

[85] Karnik R., Castelino K., Fan R., Yang P., Majumdar A. (2005). Effects of Biological Reactions and Modifications on Conductance of Nanofluidic Channels. Nano Lett..

[86] Schoch R.B., Cheow L.F., Han J. (2007). Electrical Detection of Fast Reaction Kinetics in Nanochannels with an Induced Flow. Nano Lett..

[87] Durand N.F.Y., Renaud P. (2009). Label-free determination of protein–surface interaction kinetics by ionic conductance inside a nanochannel. Lab Chip.

[88] Hsueh H.T., Lin C.T. (2016). An incremental double-layer capacitance of a planar nano gap and its application in cardiac-troponin T detection. Biosens. Bioelectron..

[89] Liao T., Li X., Tong Q., Zou K., Zhang H., Tang L., Sun Z., Zhang G. (2017). Ultrasensitive Detection of MicroRNAs with Morpholino-Functionalized Nanochannel Biosensor. Anal. Chem..

[90] Duan C., Alibakhshi M.A., Kim D.K., Brown C.M., Craik C.S., Majumdar A. (2016). Label-Free Electrical Detection of Enzymatic Reactions in Nanochannels. ACS Nano.

[91] Kim S.J., Wang Y., Lee J.H., Jang H., Han J. (2007). Concentration Polarization and Nonlinear Electrokinetic Flow near Nanofluidic Channel. Phys. Rev. Lett..

[92] Chang C., Yeh C., Yang R. (2012). Ion concentration polarization near microchannel–nanochannel interfaces: Effect of pH value. Electrophoresis.

[93] Huang K., Yang R. (2008). A nanochannel-based concentrator utilizing the concentration polarization effect. Electrophoresis.

[94] Ahmed Z., Bu Y., Yobas L. (2020). Conductance Interplay in Ion Concentration Polarization across 1D Nanochannels: Microchannel Surface Shunt and Nanochannel Conductance. Anal. Chem..

[95] Karnik R., Duan C., Castelino K., Daiguji H., Majumdar A. (2007). Rectification of Ionic Current in a Nanofluidic Diode. Nano Lett..

[96] Hou X., Yang F., Li L., Song Y., Jiang L., Zhu D. (2010). A Biomimetic Asymmetric Responsive Single Nanochannel. J. Am. Chem. Soc..

[97] Perry J.M., Zhou K., Harms Z.D., Jacobson S.C. (2010). Ion Transport in Nanofluidic Funnels. ACS Nano.

[98] Guan W., Fan R., Reed M.A. (2011). Field-effect reconfigurable nanofluidic ionic diodes. Nat. Commun..

[99] Eberle P., Höller C., Müller P., Suomalainen M., Greber U.F., Eghlidi H., Poulikakos D. (2018). Single entity resolution valving of nanoscopic species in liquids. Nat. Nanotechnol..

[100] Lin L., Mawatari K., Morikawa K., Kitamori T. (2016). Living Single Cell Analysis Platform Utilizing Microchannel, Single Cell Chamber, and Extended-nano Channel. Anal. Sci..

[101] Lin L., Mawatari K., Morikawa K., Pihosh Y., Yoshizaki A., Kitamori T. (2017). Micro/extended-nano sampling interface from a living single cell. Analyst.

[102] Peng R., Li D. (2017). Detection and sizing of nanoparticles and DNA on PDMS nanofluidic chips based on differential resistive pulse sensing. Nanoscale.

[103] Harms Z.D., Mogensen K.B., Nunes P.S., Zhou K., Hildenbrand B.W., Mitra I., Tan Z., Zlotnick A., Kutter J.P., Jacobson S.C. (2011). Nanofluidic Devices with Two Pores in Series for Resistive-Pulse Sensing of Single Virus Capsids. Anal. Chem..

[104] Kondylis P., Schlicksup C.J., Brunk N.E., Zhou J., Zlotnick A., Jacobson S.C. (2019). Competition between Normative and Drug-Induced Virus Self-Assembly Observed with Single-Particle Methods. J. Am. Chem. Soc..

[105] Yasaki H., Shimada T., Yasui T., Yanagida T., Kaji N., Kanai M., Nagashima K., Kawai T., Baba Y. (2018). Robust Ionic Current Sensor for Bacterial Cell Size Detection. ACS Sens..

[106] Pennathur S., Santiago J.G. (2005). Electrokinetic Transport in Nanochannels. 2. Experiments. Anal. Chem..

[107] Haywood D.G., Harms Z.D., Jacobson S.C. (2014). Electroosmotic Flow in Nanofluidic Channels. Anal. Chem..

[108] Peng R., Li D. (2016). Electroosmotic flow in single PDMS nanochannels. Nanoscale.

[109] Li J., Li D. (2019). Electroosmotic flow velocity in DNA modified nanochannels. J. Colloid Interface Sci..

[110] Van der Heyden F.H.J., Stein D., Dekker C. (2005). Streaming currents in a single nanofluidic channel. Phys. Rev. Lett..

[111] Van der Heyden F.H.J., Stein D., Besteman K., Lemay S.G., Dekker C. (2006). Charge inversion at high ionic strength studied by streaming currents. Phys. Rev. Lett..

[112] Van der Heyden F.H.J., Bonthuis D.J., Stein D., Meyer C., Dekker C. (2006). Electrokinetic Energy Conversion Efficiency in Nanofluidic Channels. Nano Lett..

[113] Morikawa K., Mawatari K., Kato M., Tsukahara T., Kitamori T. (2010). Streaming potential/current measurement system for investigation of liquids confined in extended-nanospace. Lab Chip.

[114] Morikawa K., Mawatari K., Kazoe Y., Tsukahara T., Kitamori T. (2011). Shift of isoelectric point in extended nanospace investigated by streaming current measurement. Appl. Phys. Lett..

[115] Morikawa K., Kazoe Y., Mawatari K., Tsukahara T., Kitamori T. (2015). Dielectric Constant of Liquids Confined in the Extended Nanospace Measured by a Streaming Potential Method. Anal. Chem..

[116] Xu Y., Xu B. (2015). An Integrated Glass Nanofluidic Device Enabling In-situ Electrokinetic Probing of Water Confined in a Single Nanochannel under Pressure-Driven Flow Conditions. Small.

[117] Siria A., Poncharal P., Biance A., Fulcrand R., Blase X., Purcell S.T., Bocquet L. (2013). Giant osmotic energy conversion measured in a single transmembrane boron nitride nanotube. Nature.

[118] Zevenbergen M.A.G., Singh P.S., Goluch E.D., Wolfrum B.L., Lemay S.G. (2011). Stochastic Sensing of Single Molecules in a Nanofluidic Electrochemical Device. Nano Lett..

[119] Kang S., Nieuwenhuis A.F., Mathwig K., Mampallil D., Lemay S.G. (2013). Electrochemical Single-Molecule Detection in Aqueous Solution Using Self-Aligned Nanogap Transducers. ACS Nano.

[120] Rassaei L., Mathwig K., Kang S., Heering H.A., Lemay S.G. (2014). Integrated Biodetection in a Nanofluidic Device. ACS Nano.

[121] Mathwig K., Mampallil D., Kang S., Lemay S.G. (2012). Electrical cross-correlation spectroscopy: Measuring picoliter-per-minute flows in nanochannels. Phys. Rev. Lett..

[122] Sanghavi B.J., Varhue W., Rohani A., Liao K., Bazydlo L.A.L., Chou C., Swami N.S. (2015). Ultrafast immunoassays by coupling dielectrophoretic biomarker enrichment in nanoslit channel with electrochemical detection on graphene. Lab Chip.

[123] Ren Y., Stein D. (2008). Slip-enhanced electrokinetic energy conversion in nanofluidic channels. Nanotechnology.

[124] Kuang C., Wang G. (2010). A novel far-field nanoscopic velocimetry for nanofluidics. Lab Chip.

[125] Kazoe Y., Iseki K., Mawatari K., Kitamori T. (2013). Evanescent Wave-Based Particle Tracking Velocimetry for Nanochannel Flows. Anal. Chem..

[126] Liu Y., Chen X., Zhang Y., Liu J. (2019). Advancing single-cell proteomics and metabolomics with microfluidic technologies. Analyst.

[127] Luo T., Fan L., Zhu R., Sun D. (2019). Microfluidic Single-Cell Manipulation and Analysis: Methods and Applications. Micromachines.

[128] Kwon T., Ko S.H., Hamel J.P., Han J. (2020). Continuous Online Protein Quality Monitoring during Perfusion Culture Production Using an Integrated Micro/Nanofluidic System. Anal. Chem..

[129] Zhou Z., Yin B., Michel J. (2015). On-chip light sources for silicon photonics. Light Sci. Appl..

